# Diagnostic Dilemma: A Patient With Pulmonary Fibrosis Who Presented for Severe Tracheal Stenosis After COVID-19 Pneumonia

**DOI:** 10.7759/cureus.38060

**Published:** 2023-04-24

**Authors:** Natalie Hubbell, Adam Aslam, Amir Khalil, Ghulam Saydain

**Affiliations:** 1 Internal Medicine, Wayne State University, Detroit, USA; 2 Internal Medicine, Wayne State University, Detroit Medical Center, Detroit, USA

**Keywords:** covid-19 pneumonia, pulmonary fibrosis, tracheostomy complications, interstitial lung disease, post-covid sequelae, covid-19, tracheal stenosis

## Abstract

A 44-year-old man with pulmonary fibrosis presented to our pulmonary hypertension clinic with biphasic stridor and dyspnea. He was sent to the emergency department, where he was found to have 90% subglottic tracheal stenosis and was successfully treated with balloon dilation. Seven months prior to the presentation, he required intubation for coronavirus disease 2019 (COVID-19) pneumonia complicated by hemorrhagic stroke. He was discharged after percutaneous dilatational tracheostomy, which was decannulated after three months.

Our patient possessed several risk factors for tracheal stenosis, including endotracheal intubation, tracheostomy, and airway infection. Furthermore, our case is of great importance given the developing literature on COVID-19 pneumonia and its subsequent complications. Additionally, his history of interstitial lung disease may have confounded his presentation. Therefore, it is important to understand stridor, as it is an important exam finding that clinically distinguishes upper and lower airway disease. Our patient’s biphasic stridor is consistent with the diagnosis of severe tracheal stenosis.

## Introduction

Throughout the coronavirus disease 2019 (COVID-19) pandemic, we continue to find a wide variety of complications associated with not just COVID-19 pneumonia but also non-pneumonia COVID-19 infection. Our case report is unique for many reasons: our case follows a young man with pulmonary fibrosis (PF) who was found to have tracheal stenosis (TS) months after endotracheal intubation (EI), percutaneous dilatational tracheostomy (PDT), and prolonged mechanical ventilation (MV). While many of these serve as independent risk factors for the development of TS, our case is important for two major reasons, which we aim to highlight in this presentation.

Firstly, stridor is often confounded with wheezing. Our patient presented with interstitial lung disease and PF with chronic diffuse wheezing and crackles on the exam. All of the aforementioned findings posed a diagnostic challenge in recognizing stridor separately from his baseline respiratory exam. Secondly, we aim to add to the growing literature on the association between TS as a complication of COVID-19.

## Case presentation

A 44-year-old man presented to our pulmonary hypertension clinic for initial evaluation for his chronic pulmonary conditions, and a chief complaint of noisy breathing. His history was relevant for systemic lupus erythematosus (SLE) and diffuse systemic sclerosis (SS), which resulted in interstitial lung disease (ILD), PF, and chronic hypoxemic respiratory failure for which he is on 2 liters of oxygen (O2) continuously.

Seven months prior to the presentation, he was hospitalized for moderate COVID-19 pneumonia, requiring increased O2 requirement above his baseline; he was discharged home with steroids. Two weeks later, he was brought to the emergency department with acute encephalopathy and was found to have a spontaneous left basal ganglia hemorrhage extending to the subarachnoid space. This spontaneous intracerebral hemorrhage (ICH) was thought to be secondary to his COVID-19 infection. He had a prolonged hospitalization wherein he underwent EI and MV for airway protection. He then had corrective neurosurgical management with craniotomy and evacuation. During the hospitalization, he had difficulty weaning off the ventilator, so he underwent PDT after two weeks of EI. He was discharged to a long-term acute care center (LTAC) on MV, wherein he was eventually weaned off the ventilator. His tracheostomy was decannulated after three months. Much of his motor function has improved, but the patient continues to have bulbar weakness from the ICH. His oxygen requirements have returned to his baseline of 2 liters of O2.

**Figure 1:**
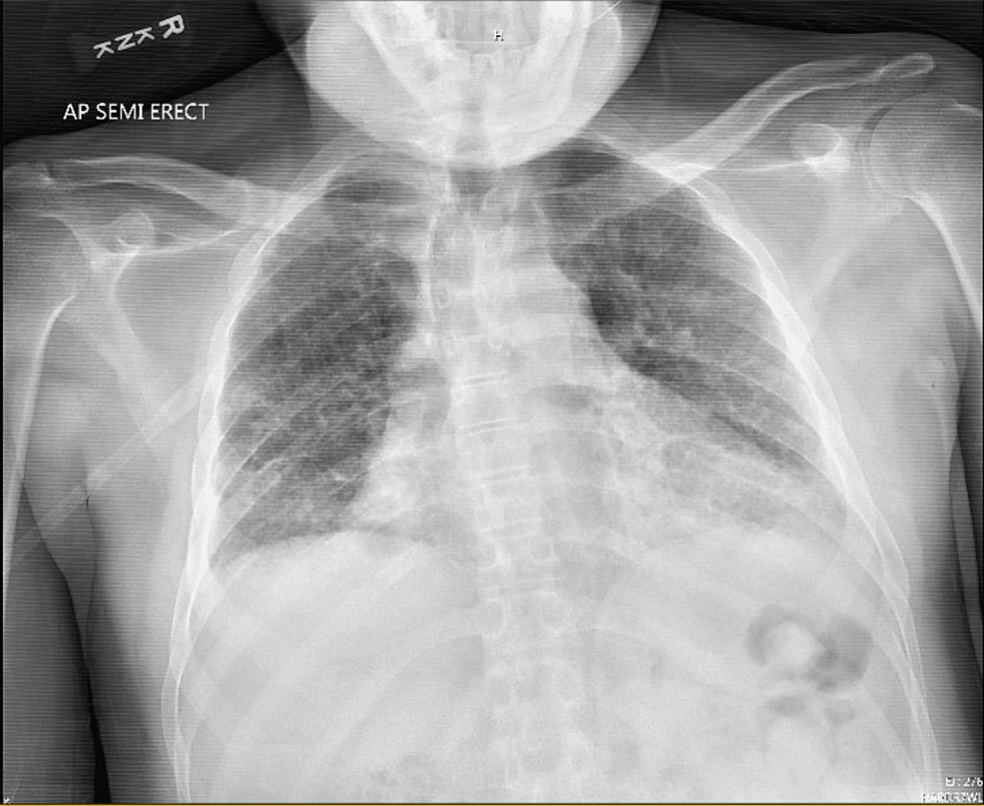
Pulmonary fibrosis and chronic obstructive pulmonary disease with basilar medial infiltrates, pneumonia, or atelectasis

**Figure 2:**
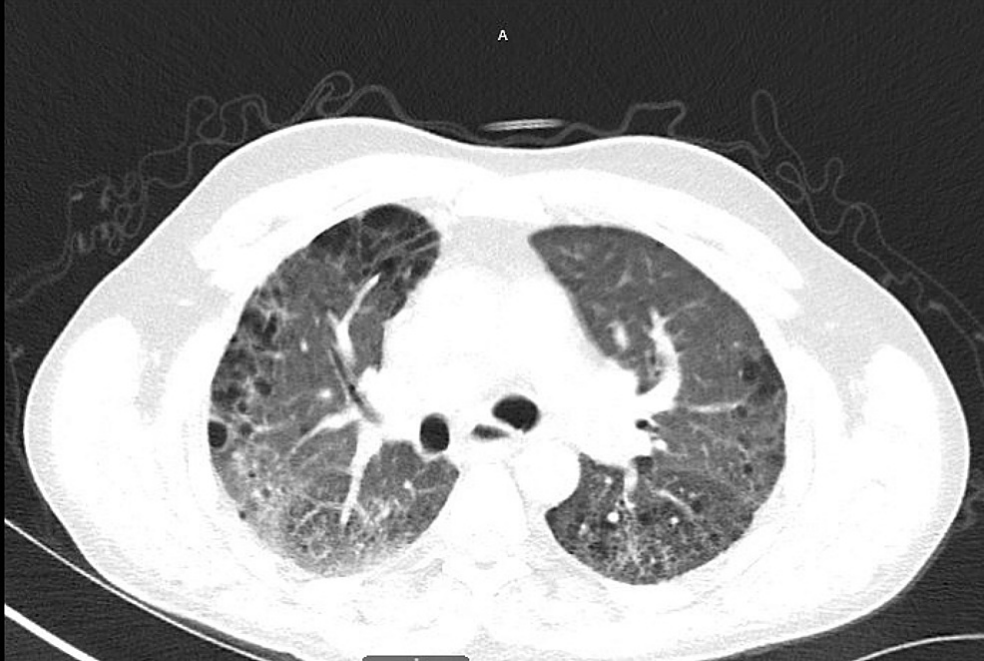
Bilateral severe pulmonary fibrosis consistent with connective tissue disease

**Figure 3:**
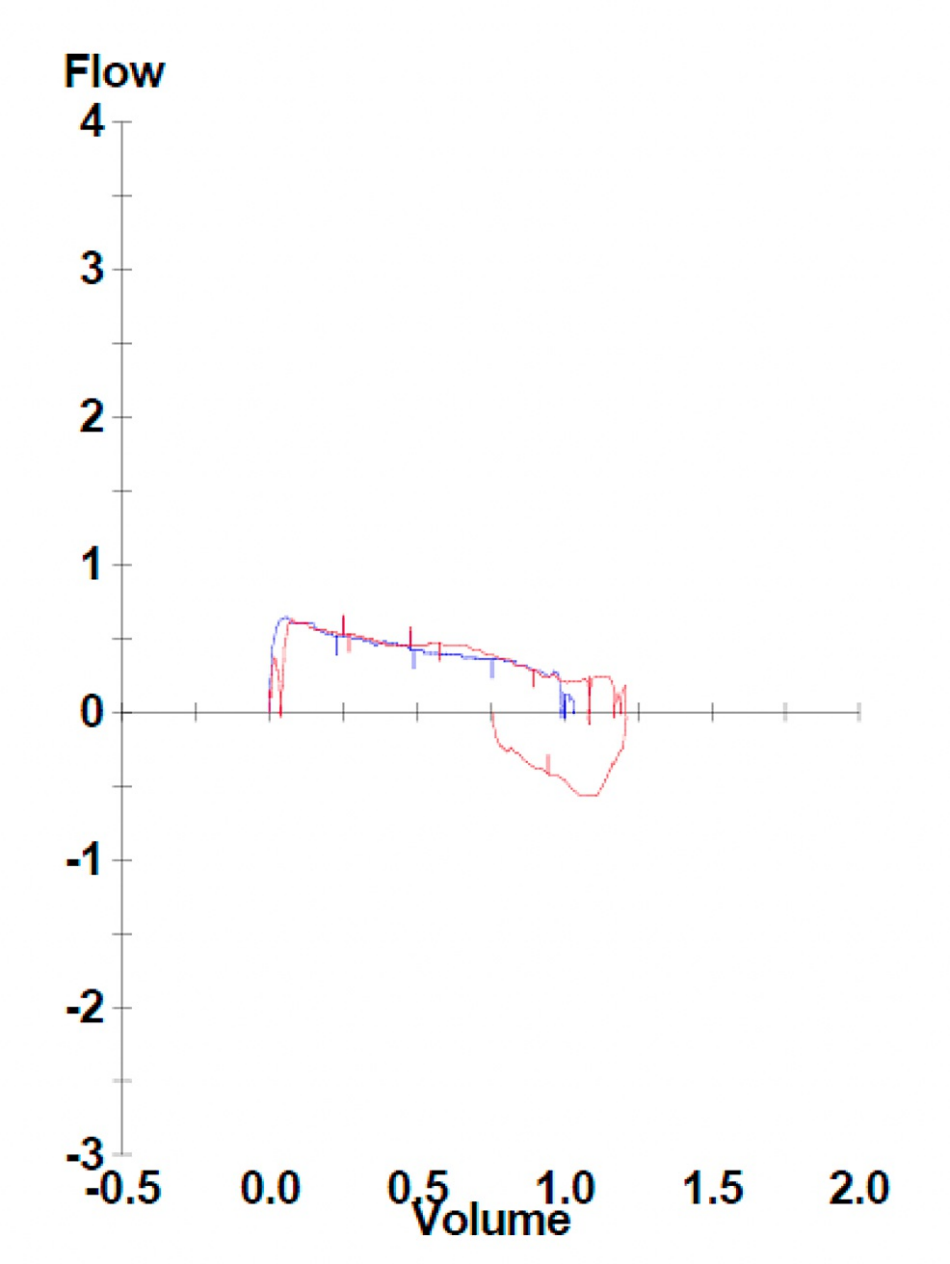
Severe obstructive ventilator defect

After returning home from the LTAC, his girlfriend noticed him to have noisy breathing that had progressively worsened over a four-month period. The patient was examined by his previous pulmonologist, who documented that he found chronic wheezing associated with his ILD, and recommended adherence to his inhalers and oxygen. His inhaler regimen consisted of budesonide-formoterol 80 mcg-4.5 mcg twice daily, ipratropium 17 mcg daily, and albuterol every six hours as needed for respiratory distress. A chest X-ray and thoracic CT demonstrate PF, as seen in Figures 1, 2, respectively. The patient had pulmonary function testing (PFT) to assess the severity of his chronic obstructive pulmonary disease. His PFT was pertinent for severe obstructive ventilator defect, as visualized with his flow volume loop in Figure 3.

Due to persistent noisy breathing, he presented to our clinic. While in triage, he was found to have mild respiratory distress and audible stridor. On exam, he was found to be hemodynamically stable and saturating 100% on his baseline 2 liters of O2. His lung exam revealed diffuse dry crackles and expiratory wheezing in all lung fields. Auscultation of the neck revealed biphasic stridor that was notably distinct from his wheezing. While his speech was labored, he did not have any muffling of his voice, drooling, or pooling of fluid in the oropharynx. His tongue and uvula were midline, and he had a visible scar from tracheostomy decannulation in his midline neck 3 to 4 centimeters superior to his sternoclavicular joint.

Due to his respiratory distress with audible stridor, our concern for acute or subacute upper airway disease was high, so he was transferred to the emergency department where he was further evaluated.

Investigations

**Figure 4:**
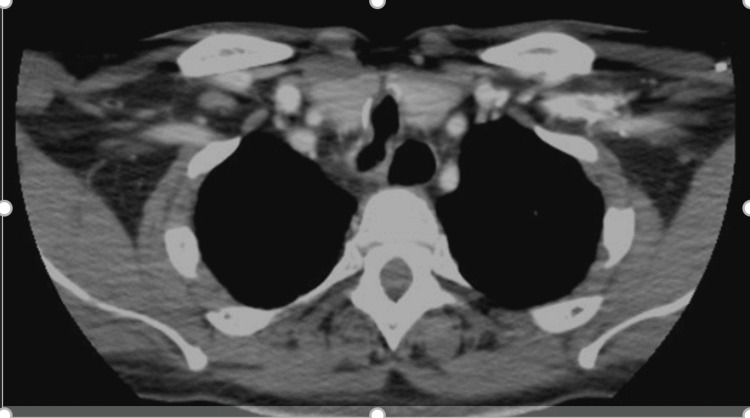
Moderate narrowing of the subglottic airway with adjacent surgical clips at the site of the prior tracheostomy

In the emergency department, our patient was evaluated by an otolaryngologist. The otolaryngologist attempted to evaluate the patient at the bedside with flexible bronchoscopy, but due to tracheal narrowing, he was unable to advance the probe beyond the area of narrowing. Therefore, the patient underwent a CT of the neck, which demonstrated moderate-sized narrowing of the subglottic airway (Figure 4).

Differential diagnosis

Our main differential diagnosis for our patient’s stridor included TS, tracheal foreign body, or extra-laryngeal mass. These were our initial differential diagnoses as he presented with biphasic stridor. This suggested a fixed lesion, either externally or internally to the upper airway, that caused stridor both during inspiration and expiration. Other less likely diagnoses included tracheomalacia, tracheitis, or vocal cord paralysis; however, the patient’s pattern of stridor, tracheomalacia, or tracheitis would present primarily with expiratory stridor; furthermore, tracheitis would be unlikely to persist for several months without the presence of other signs of infection. Vocal cord paralysis presents primarily as inspiratory stridor, given the lesion exists within the airway and at the level of the vocal cords. The pathophysiology of stridor is explained in the discussion section.

Treatment

**Figure 5:**
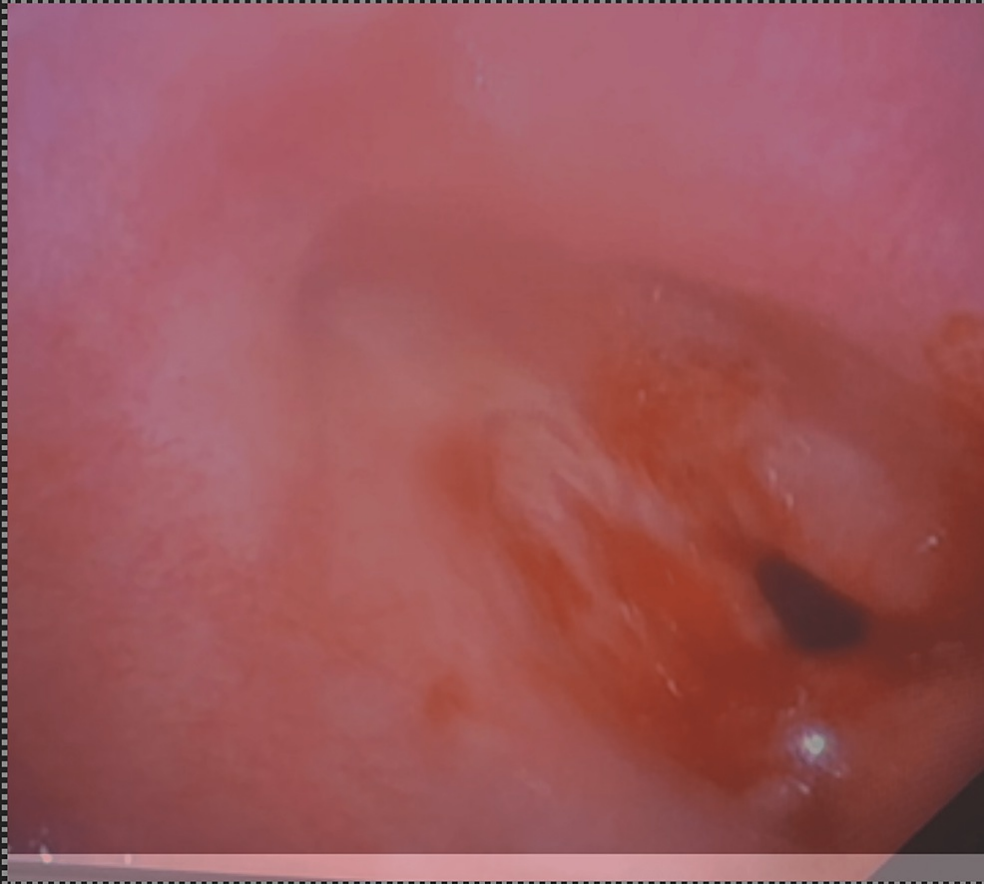
Direct laryngoscopy demonstrates 90% stenosis at the level of the trachea

**Figure 6:**
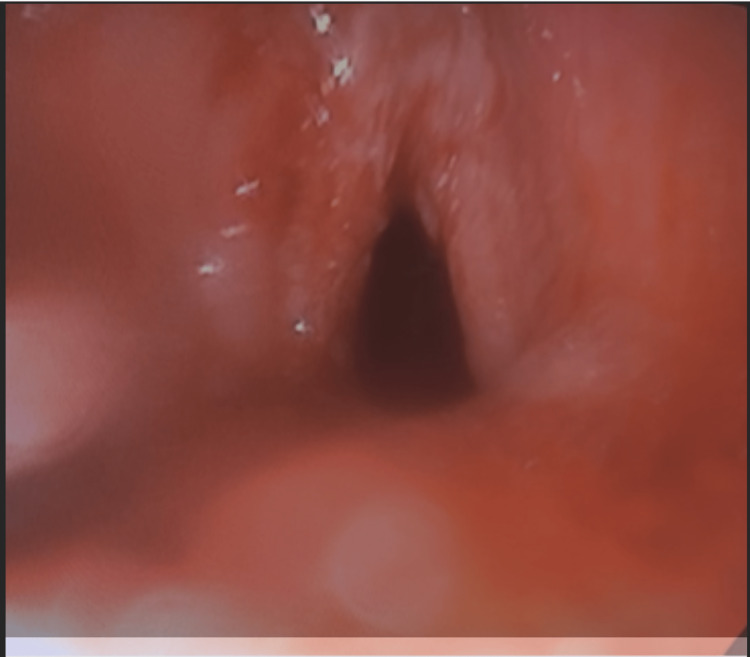
Direct laryngoscopy after balloon dilation demonstrates liberation of the stenosis to 30%

Until a definitive operation by otolaryngology, the patient was medically managed with steroids to reduce upper airway swelling, and scheduled albuterol nebulization to improve airway patency. Definitive management was established during direct laryngoscopy with laryngoscopic balloon dilation, resulting in the liberation of the stenosis from 90% to 30% (Figures 5, 6). His stridor and dyspnea resolved after dilation. He was thereafter discharged with close follow-up.

## Discussion

The main physical exam finding that clued us toward an upper airway pathology was stridor. Stridor is a unique exam finding that is found in both acute and chronic disorders of the upper airway [1]. Stridor is distinctly different from wheezing, which is a lower airway sound. While both are high-pitched sounds that occur during different phases of breathing, stridor can be recognized by auscultating the neck as opposed to the lungs. Stridor occurs because of dynamic changes to the upper airway both above and below the vocal cords and can be characterized as inspiratory, expiratory, or mixed/biphasic. Inspiratory stridor occurs in extrathoracic or supraglottic structures because the negative thoracic pressures during inspiration cause a collapse of the supraglottic structures that are not reinforced by tracheal cartilage [1]. This occurs in anaphylaxis, angioedema, vocal cord paralysis, and supraglottic infections like croup. In the expiratory stridor, which occurs in intrathoracic or subglottic structures, the positive intrathoracic pressure during expiration causes the collapse of the damaged subglottic structures [2]. This occurs in conditions like tracheitis or tracheomalacia [3]. Biphasic stridor occurs because of a fixed lesion that exists during all phases of respiration, such as with foreign body ingestion, laryngeal tumors, or, as in our patient, TS [4]. Table 1 contains a more comprehensive list of differential diagnoses based on the type of stridor.

**Table 1:** Differential diagnosis associated with different types of stridor: inspiratory, expiratory, and biphasic

Inspiratory stridor (extrathoracic)	Expiratory stridor (intrathoracic)	Biphasic stridor (fixed lesion)
Acute epiglottitis, anaphylaxis, croup, laryngomalacia, peritonsillar abscess, retropharyngeal abscess, tracheitis, vocal cord paralysis	Anaphylaxis, tracheal stenosis, tracheitis	Foreign bodies, hemangioma, tracheal stenosis, tumor/malignancy

TS is an abnormal narrowing of the tracheal lumen and may occur at a level between the cricoid cartilage and carina [5]. TS may be congenital or acquired. While acquired TS may be caused by external trauma, infections, or space-occupying lesions such as tumors, it is most commonly caused by EI and PDT. The stenosis occurs as early granulation tissue hardens into a fibrotic scar. Early intervention is key to procuring favorable outcomes. Symptoms associated with TS include dyspnea, stridor, hoarseness, and cough. Diagnosis can be established with a laryngoscope, but in cases where TS results in too significant a stenosis for the scope to pass, a CT scan of the neck can be used to aid in diagnosis. Treatment interventions include mechanical dilation by rigid bronchoscope, balloon dilation, laser resection, tracheal stenting, or injection of steroids [6]. Newer methods utilize flexible bronchoscopy [7].

The overall incidence of TS is not well studied, but some studies have defined the incidence based on the etiology. TS was found to be associated with up to 10% of patients who had a PDT, and up to 20% of patients who had EI [8,9]. One study described risk factors that contributed to the development of TS, including prolonged duration of EI (especially greater than 10 days), prolonged duration of MV after PDT, presence of airway infection, and diabetes [10]. The incidence is not clearly defined in patients with COVID-19, but several smaller studies do find an association between prolonged COVID-related hospitalizations and the development of TS [11-13]. It is difficult to conclude that COVID-19 infection causes direct tracheal injury, as many of the findings associated with COVID-19 are also implicated in the development of TS, such as the presence of airway infection, or the need for EI and PDT. However, there is one case series that histologically examines subglottic tissue samples in patients with known TS after COVID-19 infection, and found them to have multinucleated syncytial cells with prominent nucleoli, a histological finding specific to SARS-CoV-2 infection [14]. This may suggest that COVID-19 is an independent risk factor in the development of TS, but research on this topic is limited.

## Conclusions

The case demonstrates many important learning points for the recognition of airway disease. The first of these is the recognition of the stridor and the utility of describing the stridor to refine a differential diagnosis. A second learning point is the recognition of risk factors for the development of TS. These include EI, prolonged intubation, PDT, and airway infection, all of which were seen in our patient. Finally, this case reminds clinicians to consider TS in patients who had COVID-19 infection, especially in those who share the above risk factors.
